# Supplementary material for: Partitioning and aggregating cross-tissue and tissue-specific genetic effects to identify gene-trait associations
Source: Nat Commun. 2024 Jul 9;15:5769. doi: 10.1038/s41467-024-49924-4 (PMC11233643; doi:10.1038/s41467-024-49924-4)
Supplement: Supplementary file 1 — Supplementary Information [file 41467_2024_49924_MOESM1_ESM.pdf]

Supplementary Information for "Partitioning and aggregating cross-tissue and tissue-specific genetic effects to identify gene-trait associations"

Shuang Song[1], Lijun Wang[2], Lin Hou[1,3,*] , and Jun S. Liu[4,*]

[1] *Center for Statistical Science, Department of Industrial Engineering, Tsinghua University, Beijing 100084, China*

[2] *Department of Biostatistics, Yale School of Public Health, New Haven, CT 06510, USA*

[3] *MOE Key Laboratory of Bioinformatics, School of Life Sciences, Tsinghua University, Beijing 100084, China*

[4] *Department of Statistics, Harvard University, Cambridge, MA 02138, USA*

[*] *To whom correspondence should be addressed.*

1 Supplementary Figures

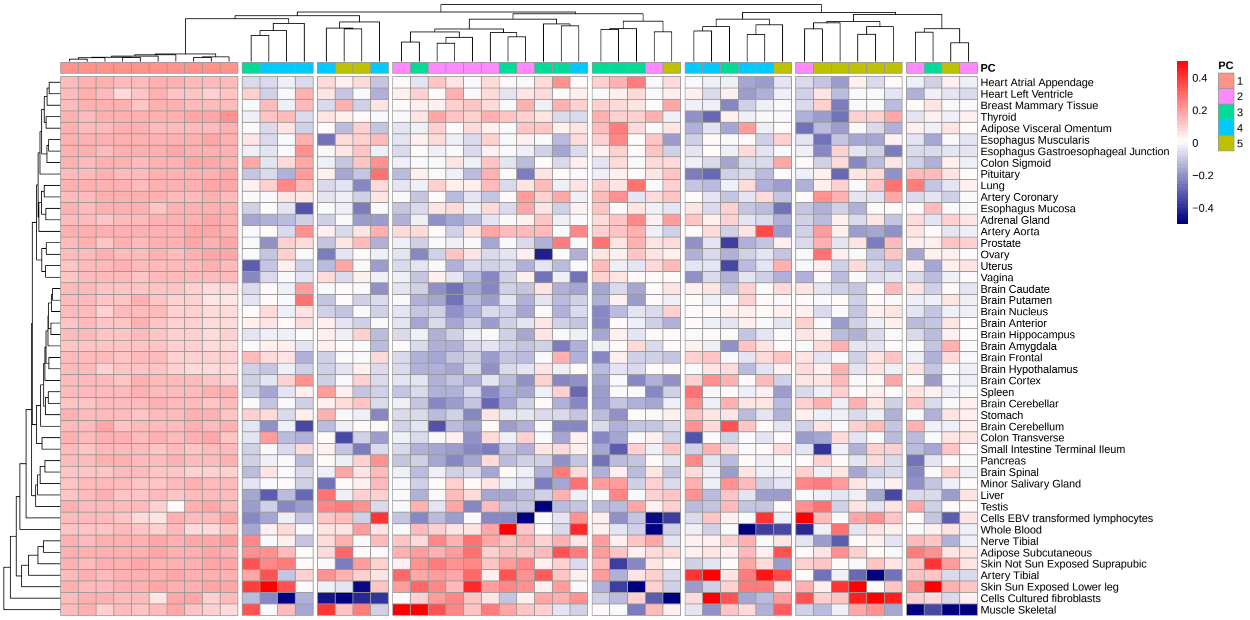

Supplementary Figure 1: **The heatmap of PCA loadings of the first 5 PCs in the 10 most predictable genes across 47 GTEx tissues.** Source data are provided as a Source Data file.

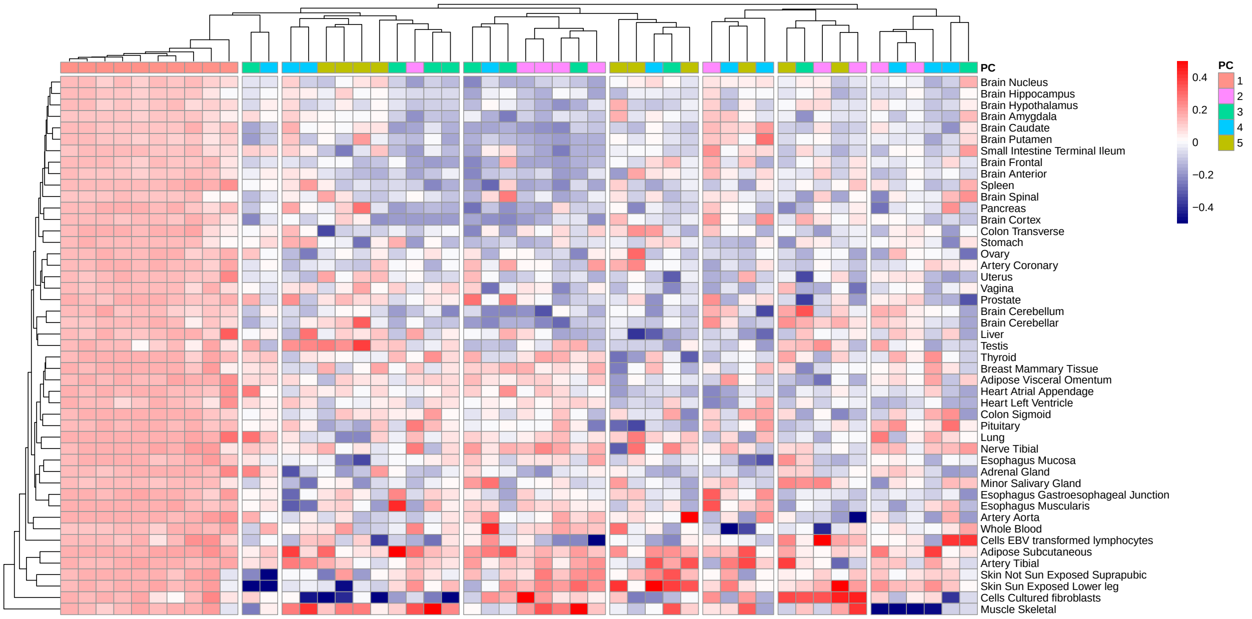

Supplementary Figure 2: **The heatmap of PCA loadings of the first 5 PCs in the 5 most predictable genes in GTEx whole blood tissue.** Source data are provided as a Source Data file.

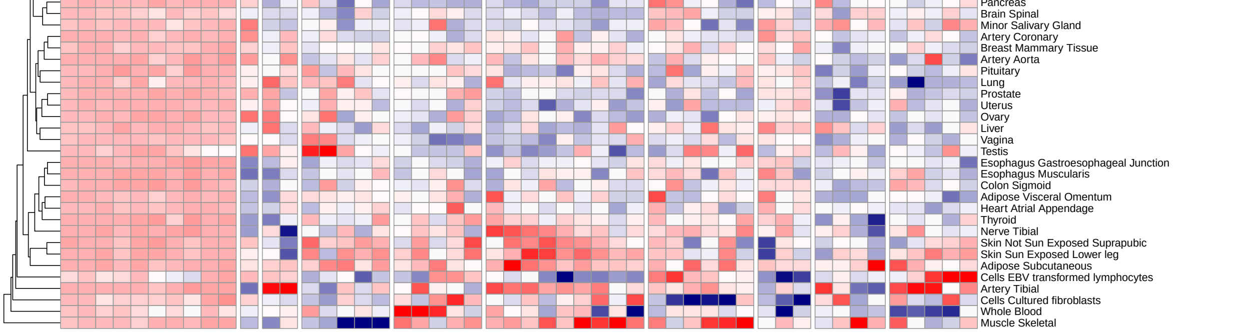

Supplementary Figure 3: **The heatmap of PCA loadings of the first 5 PCs in the 5 most predictable genes in GTEx brain cortex tissue.** Source data are provided as a Source Data file.

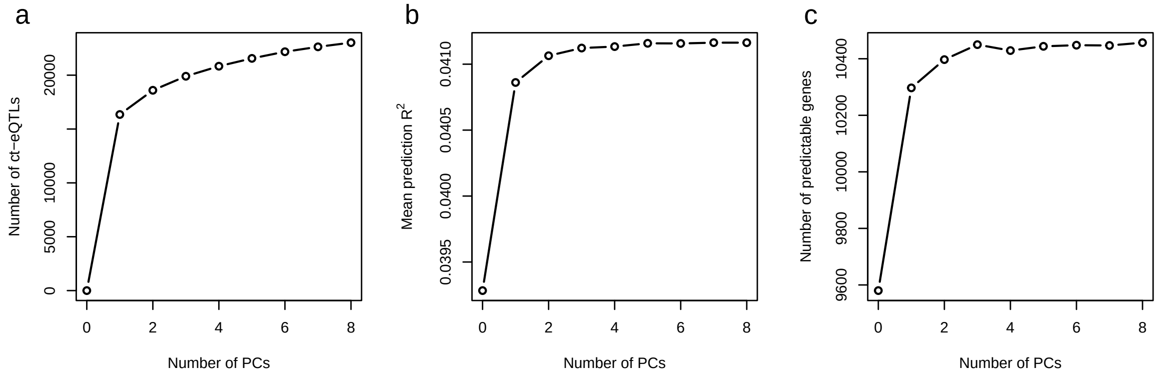

Supplementary Figure 4: **The performance of ct-eQTLs identified with the varying number of principal components (PCs).** (a) The number of ct-eQTLs identified with increasing numbers of PCs. (b) Mean prediction R^2 on the gene expression of GTEx whole blood tissue with ct-eQTLs identified with increasing PCs. (c) The number of predictable genes (prediction $R^2 > 0.01$) on GTEx whole blood tissue with ct-eQTLs identified with increasing PCs. The prediction accuracy was evaluated with a 5-fold CV. Source data are provided as a Source Data file.

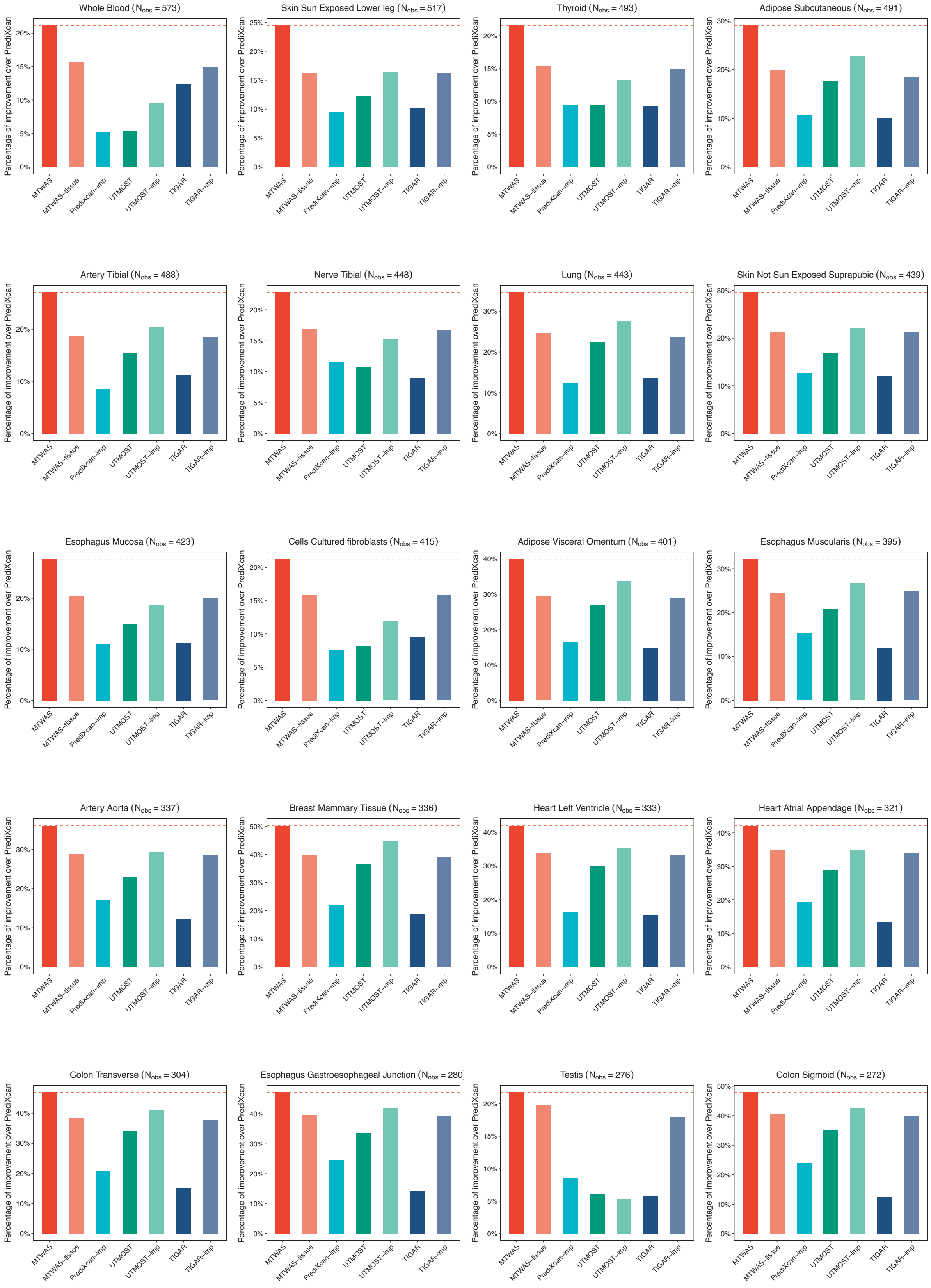
Whole Blood (N_{obs} = 573)
Skin Sun Exposed Lower leg (N_{obs} = 517)
Thyroid (N_{obs} = 493)
Adipose Subcutaneous (N_{obs} = 491)
Artery Tibial (N_{obs} = 488)
Nerve Tibial (N_{obs} = 448)
Lung (N_{obs} = 443)
Skin Not Sun Exposed Suprapubic (N_{obs} = 439)
Esophagus Mucosa (N_{obs} = 423)
Cells Cultured fibroblasts (N_{obs} = 415)
Adipose Visceral Omentum (N_{obs} = 401)
Esophagus Muscularis (N_{obs} = 395)
Artery Aorta (N_{obs} = 337)
Breast Mammary Tissue (N_{obs} = 336)
Heart Left Ventricle (N_{obs} = 333)
Heart Atrial Appendage (N_{obs} = 321)
Colon Transverse (N_{obs} = 304)
Esophagus Gastroesophageal Junction (N_{obs} = 280
Testis (N_{obs} = 276)
Colon Sigmoid (N_{obs} = 272)
Percentage of improvement over PrediXcan
MTWAS
MTWAS–tissue
PrediXcan–imp
UTMOST
UTMOST–imp
TIGAR
TIGAR–imp

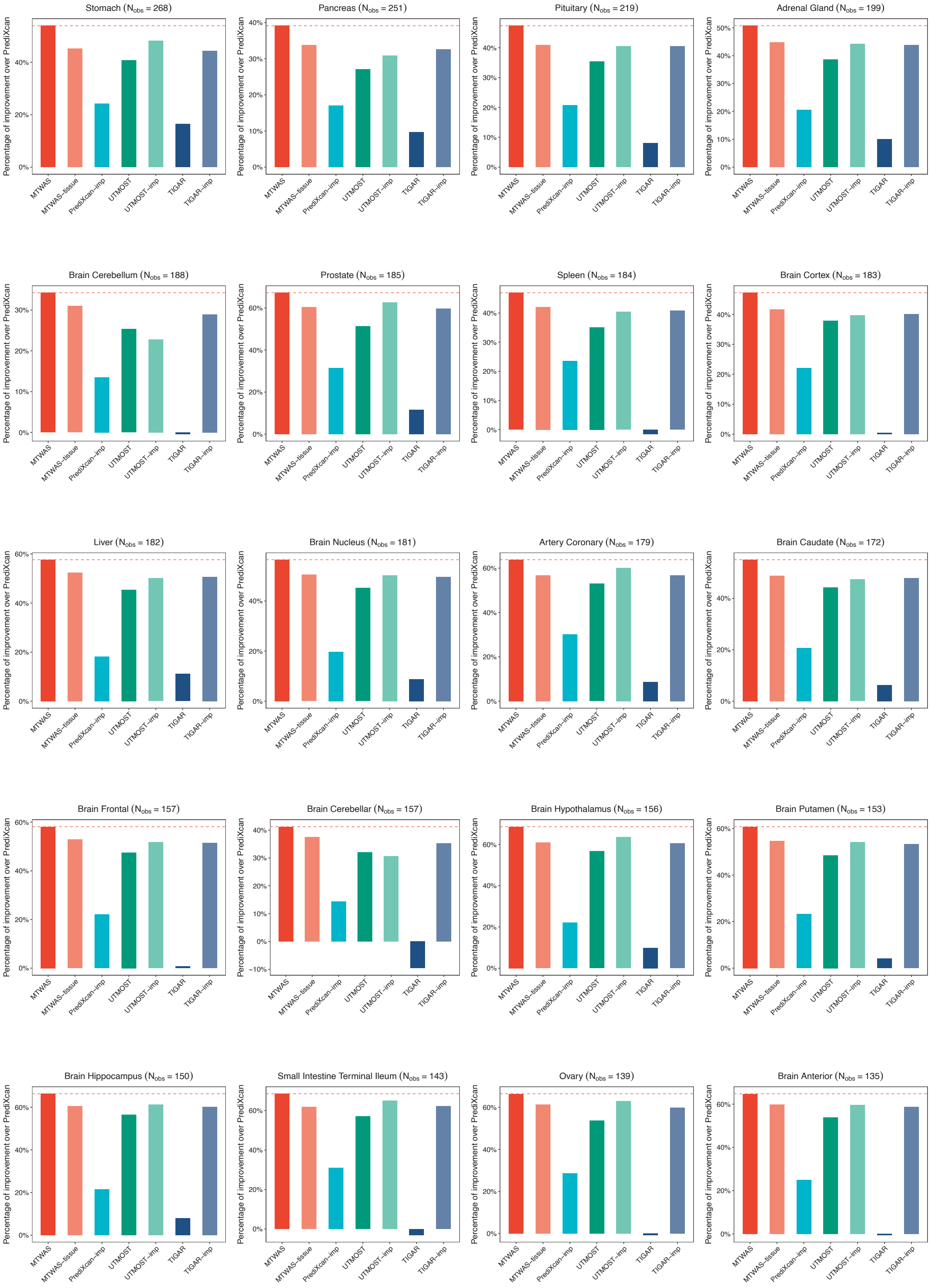
Stomach (N_{obs} = 268)
Pancreas (N_{obs} = 251)
Pituitary (N_{obs} = 219)
Adrenal Gland (N_{obs} = 199)
Brain Cerebellum (N_{obs} = 188)
Prostate (N_{obs} = 185)
Spleen (N_{obs} = 184)
Brain Cortex (N_{obs} = 183)
Liver (N_{obs} = 182)
Brain Nucleus (N_{obs} = 181)
Artery Coronary (N_{obs} = 179)
Brain Caudate (N_{obs} = 172)
Brain Frontal (N_{obs} = 157)
Brain Cerebellar (N_{obs} = 157)
Brain Hypothalamus (N_{obs} = 156)
Brain Putamen (N_{obs} = 153)
Brain Hippocampus (N_{obs} = 150)
Small Intestine Terminal Ileum (N_{obs} = 143)
Ovary (N_{obs} = 139)
Brain Anterior (N_{obs} = 135)
Percentage of improvement over PrediXcan
MTWAS
MTWAS–tissue
PrediXcan–imp
UTMOST
UTMOST–imp
TIGAR
TIGAR–imp

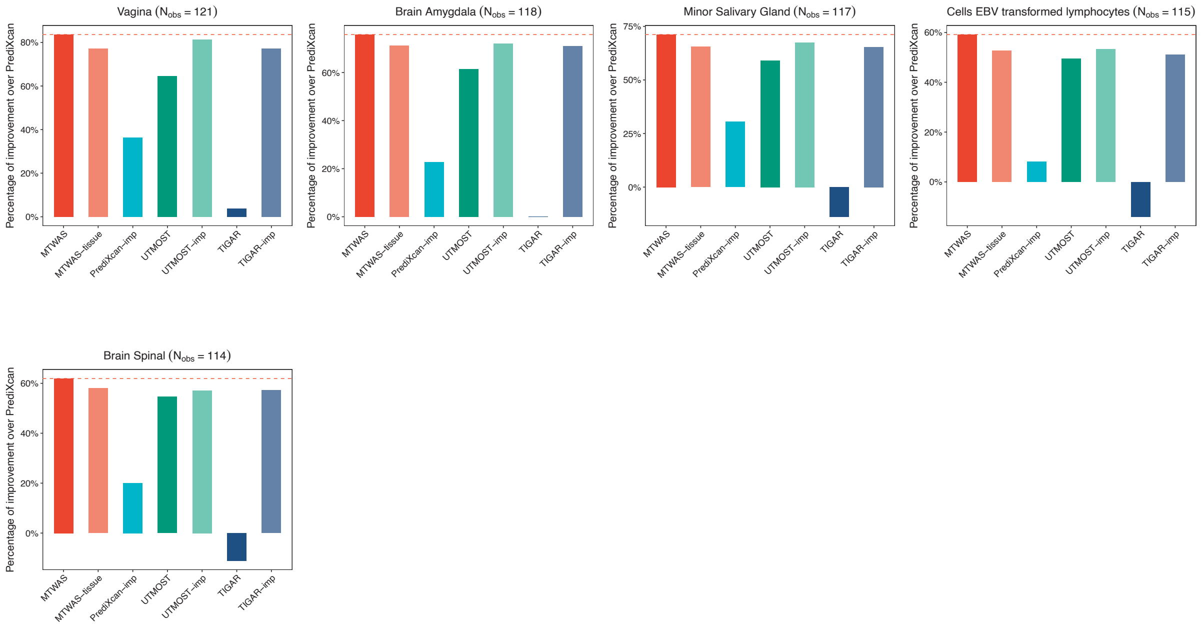

Supplementary Figure 5: **Improvements of prediction R^2 over PrediXcan, of MTWAS, MTWAS-tissue, PrediXcan-imp, UTMOST, UTMOST-imp, TIGAR (DPR), and TIGAR-imp, evaluated on GTEx datasets.** The dashed line marks the performance of MTWAS. The tissues are arranged in descending order based on their sample sizes. The prediction R^2 is based on 5-fold CV. Source data are provided as a Source Data file.

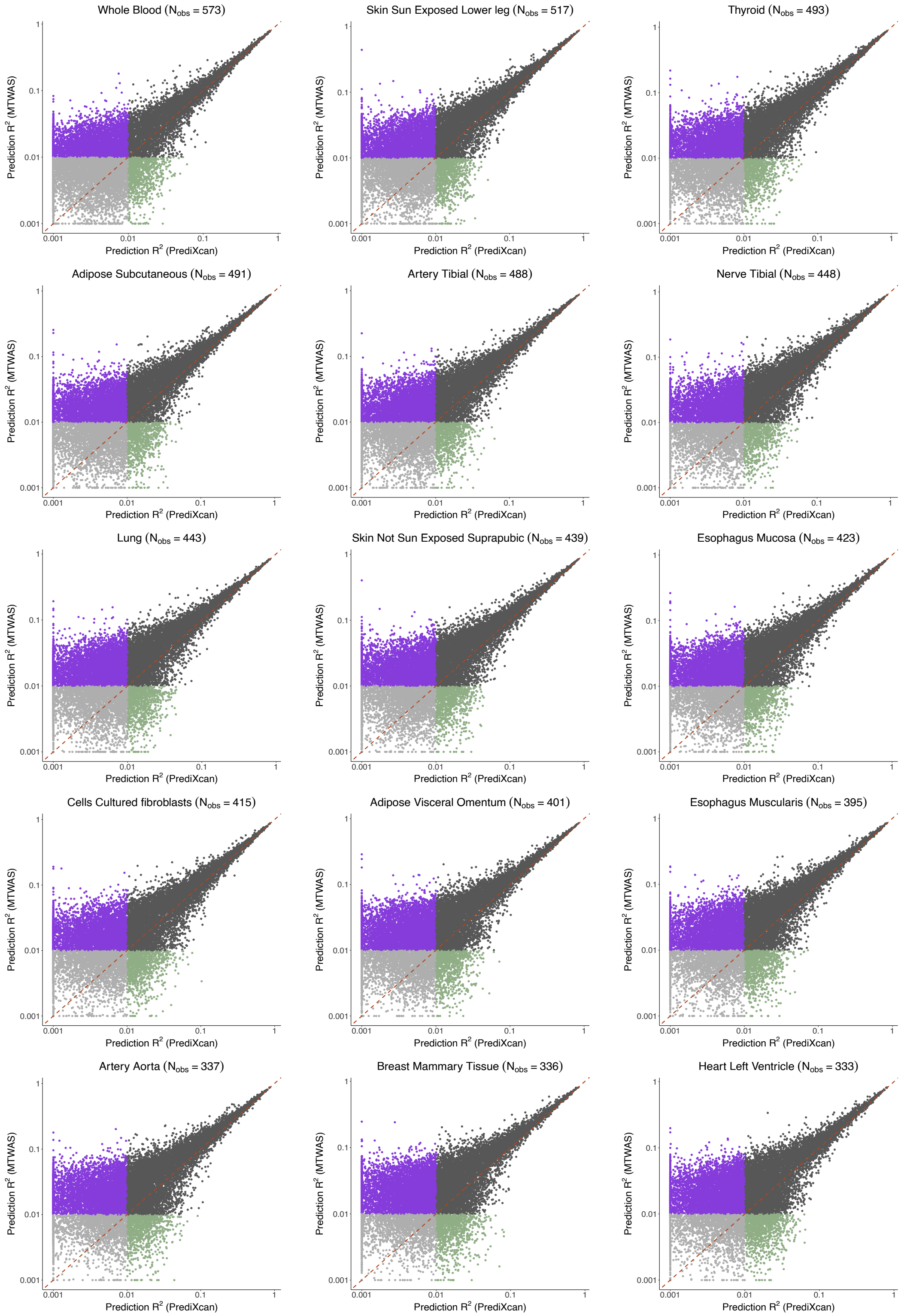
Whole Blood ($N_{obs} = 573$)
Skin Sun Exposed Lower leg ($N_{obs} = 517$)
Thyroid ($N_{obs} = 493$)
Adipose Subcutaneous ($N_{obs} = 491$)
Artery Tibial ($N_{obs} = 488$)
Nerve Tibial ($N_{obs} = 448$)
Lung ($N_{obs} = 443$)
Skin Not Sun Exposed Suprapubic ($N_{obs} = 439$)
Esophagus Mucosa ($N_{obs} = 423$)
Cells Cultured fibroblasts ($N_{obs} = 415$)
Adipose Visceral Omentum ($N_{obs} = 401$)
Esophagus Muscularis ($N_{obs} = 395$)
Artery Aorta ($N_{obs} = 337$)
Breast Mammary Tissue ($N_{obs} = 336$)
Heart Left Ventricle ($N_{obs} = 333$)
Prediction R^2 (MTWAS)
Prediction R^2 (PrediXcan)
0.001
0.01
0.1
1

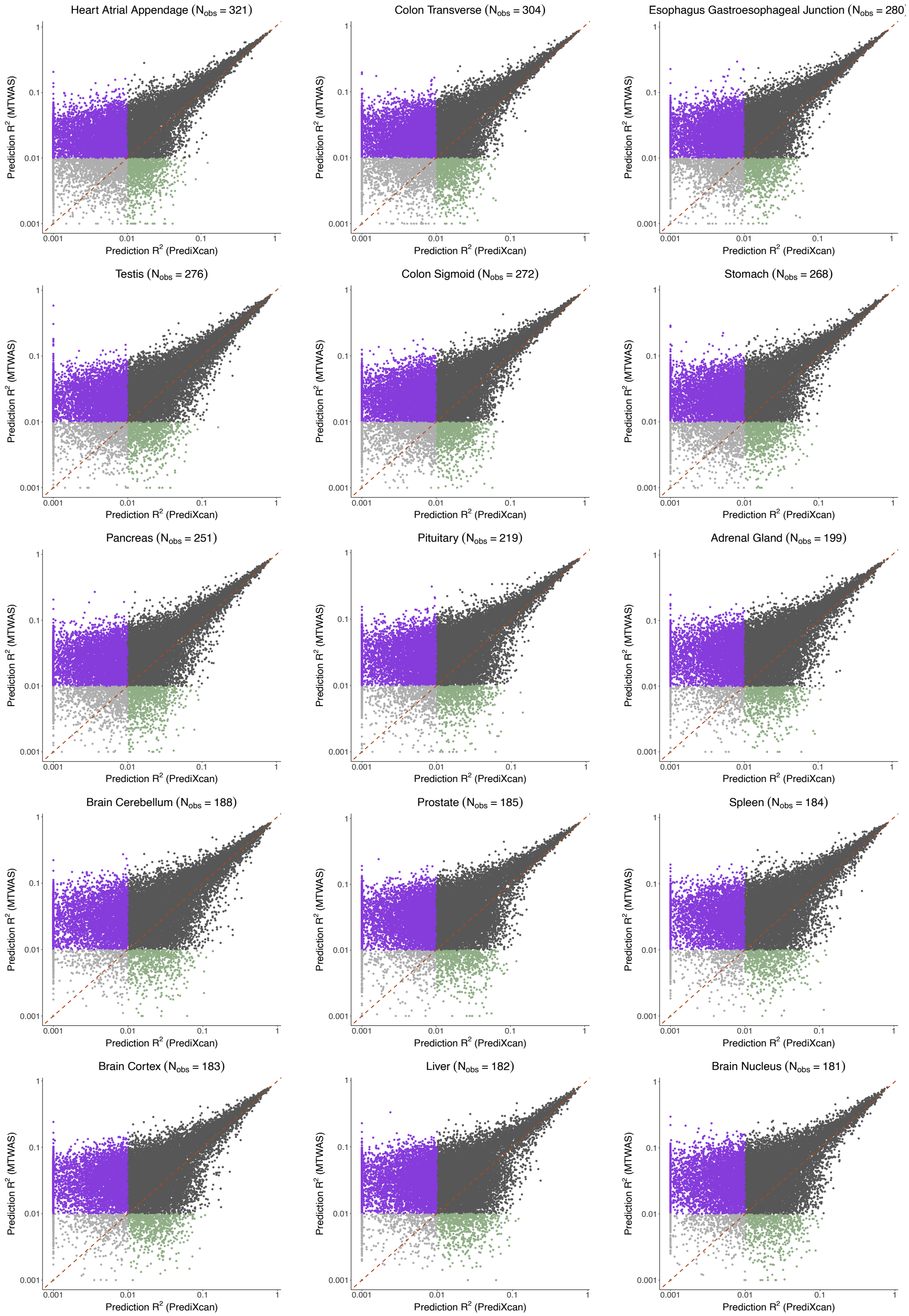

Heart Atrial Appendage (N_{obs} = 321)
Colon Transverse (N_{obs} = 304)
Esophagus Gastroesophageal Junction (N_{obs} = 280
Testis (N_{obs} = 276)
Colon Sigmoid (N_{obs} = 272)
Stomach (N_{obs} = 268)
Pancreas (N_{obs} = 251)
Pituitary (N_{obs} = 219)
Adrenal Gland (N_{obs} = 199)
Brain Cerebellum (N_{obs} = 188)
Prostate (N_{obs} = 185)
Spleen (N_{obs} = 184)
Brain Cortex (N_{obs} = 183)
Liver (N_{obs} = 182)
Brain Nucleus (N_{obs} = 181)
Prediction R^2 (MTWAS)
Prediction R^2 (PrediXcan)
0.001
0.01
0.1
1

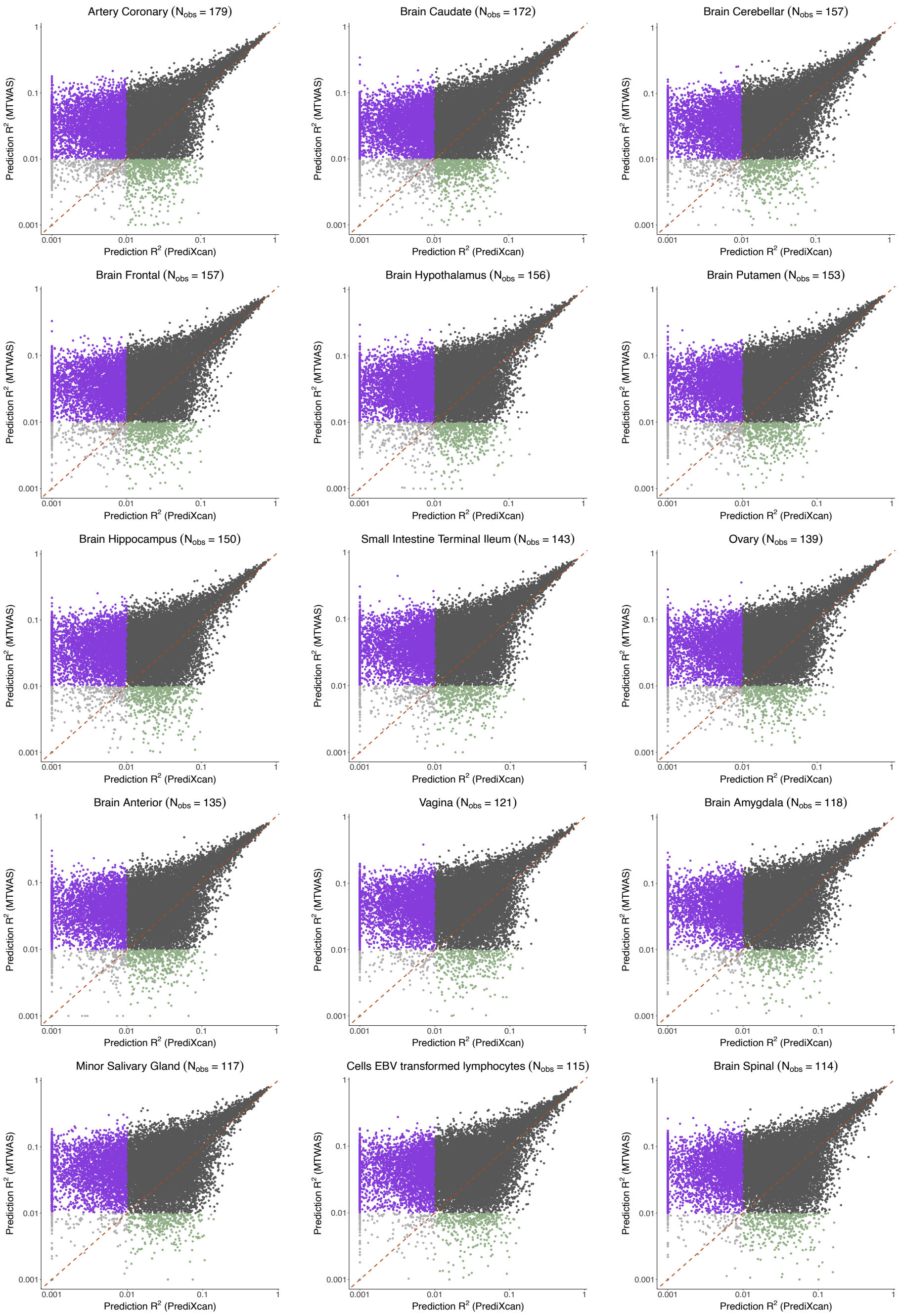

Supplementary Figure 6: **A comparison between the prediction accuracy of MTWAS with that of PrediXcan on the GTEx datasets.** The performances were evaluated on 45 GTEx tissues. The results of the other 2 tissues are in the main text. The x-axis and y-axis represent the prediction R^2 derived from a 5-fold cross-validation. Purple and green dots represent genes that have prediction $R^2 > 0.01$ *only* for MTWAS and PrediXcan, respectively. Darker and shallower grey dots represent genes that consistently have prediction $R^2 > 0.01$ and $R^2 \leq 0.01$, respectively, using both methods. Source data are provided as a Source Data file.

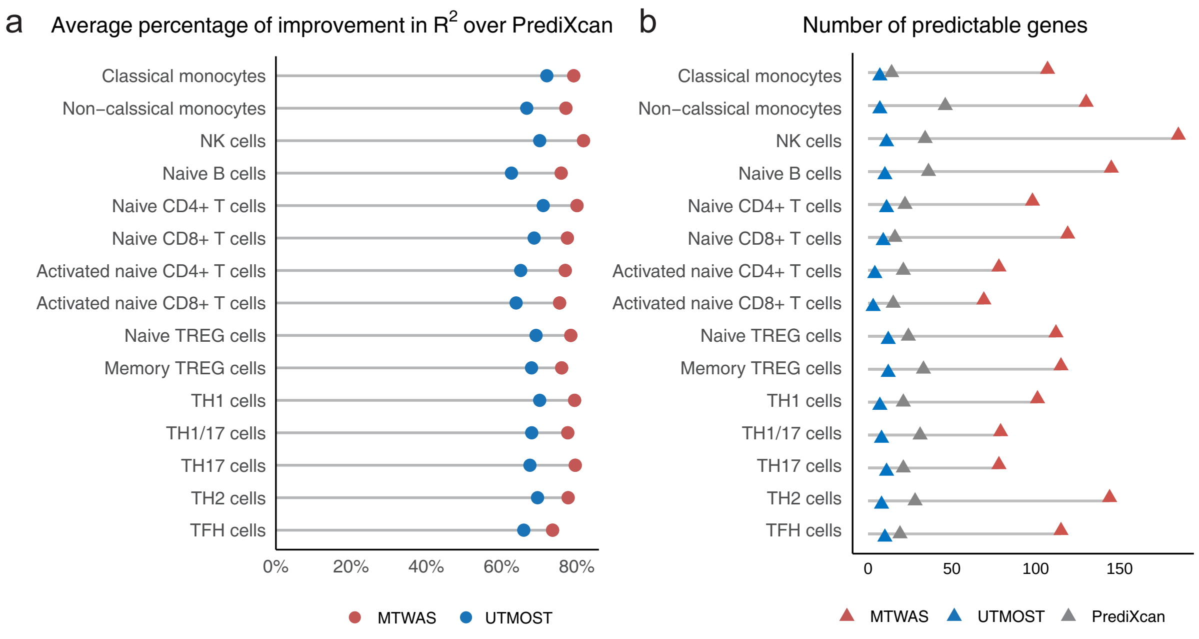

Supplementary Figure 7: **The prediction accuracy on 13 types of immune cells and 2 activation conditions of the DICE dataset.** **a**, The improvement of prediction R^2 over PrediXcan, of MTWAS and UTMOST. The prediction R^2 is based on 5-fold CV. **b**, The number of predictable genes by the three methods ($FDR < 0.05$). Source data are provided as a Source Data file.

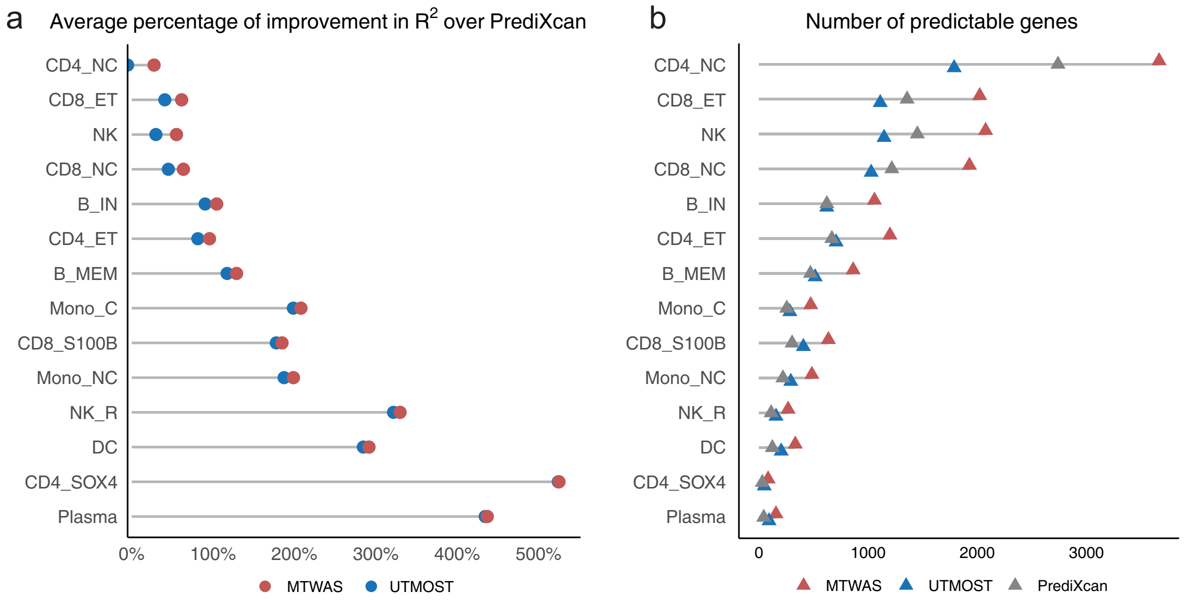

Supplementary Figure 8: **The prediction accuracy on 14 immune cell types of the OneK1K dataset. a**, The improvement of prediction R^2 over PrediXcan, of MTWAS and UTMOST. The prediction R^2 is based on 5-fold CV. **b**, The number of predictable genes by the three methods ($FDR < 0.05$). The cell types are arranged in descending order based on the total cell counts, from the largest (CD4 NC, $N_{cell} = 463,528$) to the smallest (Plasma, $N_{cell} = 3,625$). Source data are provided as a Source Data file.

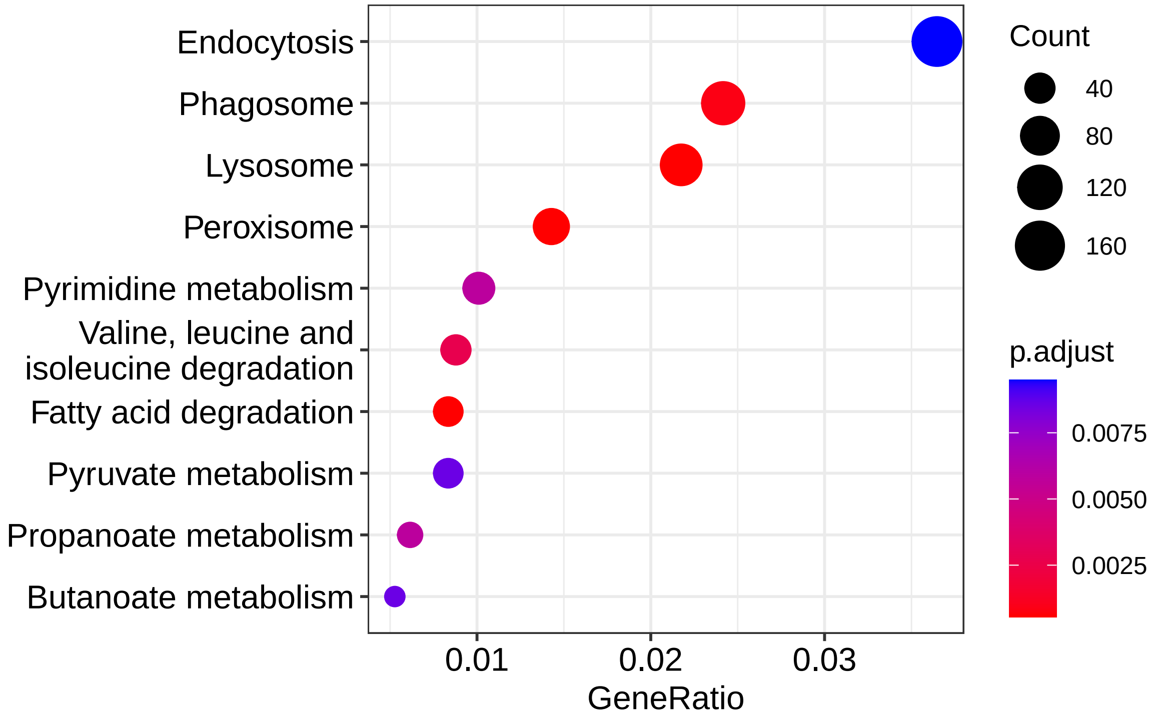

Supplementary Figure 9: **KEGG pathway enrichment analysis of cross-tissue genes (genes that are regulated only by ct-eQTLs).** The significance of enrichment is depicted through the color of the dots, and the gene ratio is indicated by the horizontal distance between the dots and the y-axis. The sizes of the dots correspond to the gene counts. Source data are provided as a Source Data file.

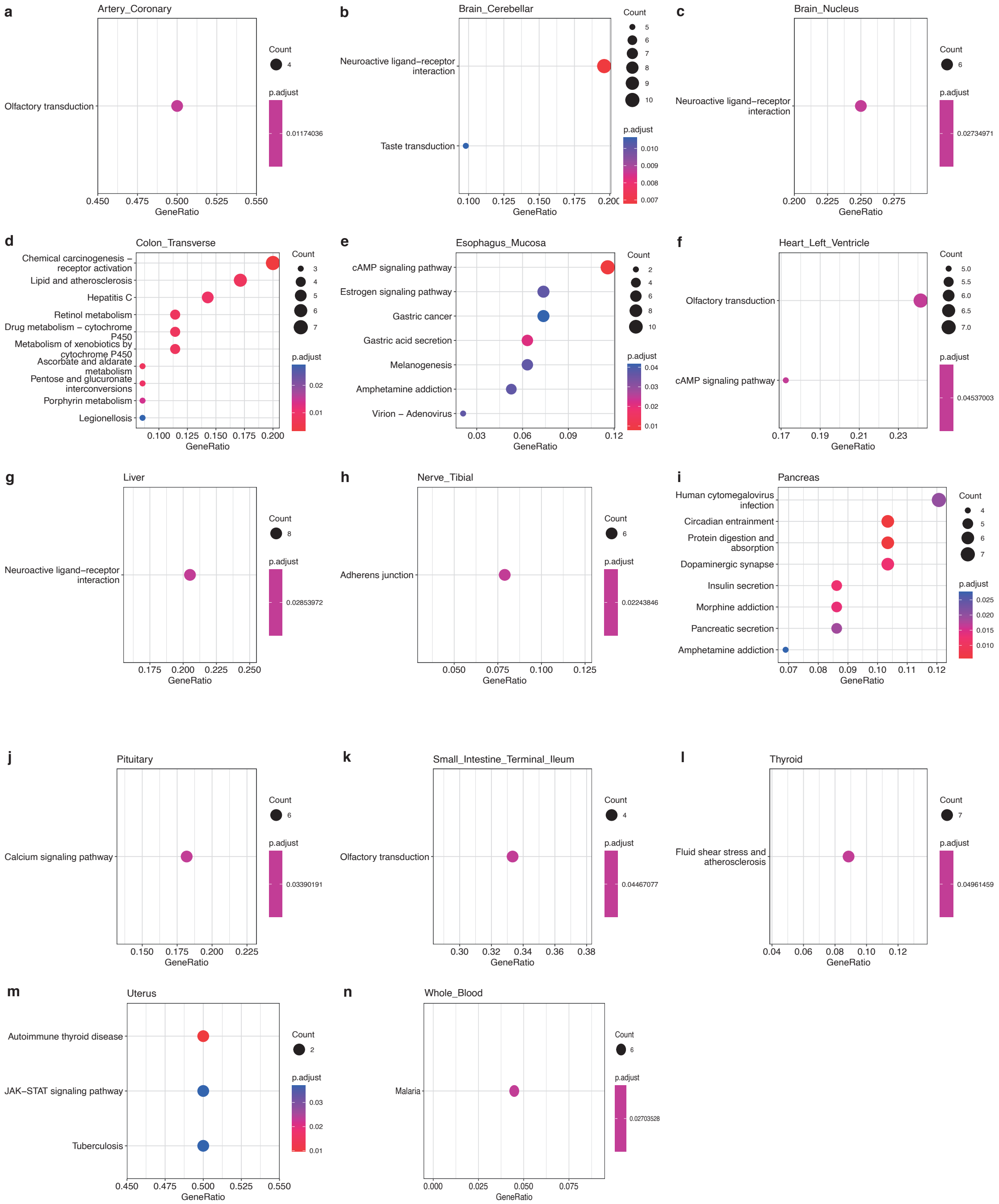

Supplementary Figure 10: **KEGG pathway enrichment analysis of tissue-specific genes (genes that are regulated only by ts-eQTLs).** The significance of enrichment is depicted through the color of the dots, and the gene ratio is indicated by the horizontal distance between the dots and the y-axis. The sizes of the dots correspond to the gene counts. Panels **a-n** correspond to 14 GTEx tissues with significantly enriched pathways in tissue-specific genes (adjusted $p < 0.05$). Source data are provided as a Source Data file.

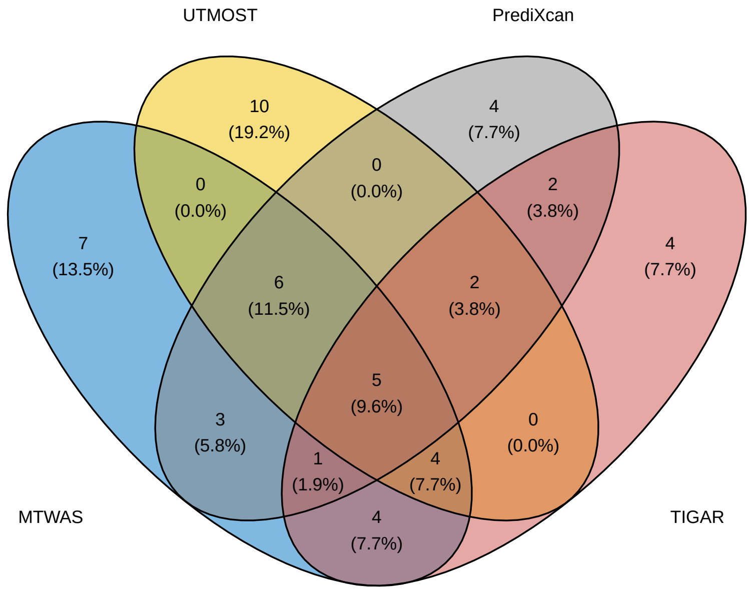

Supplementary Figure 11: **Venn diagram of genes significantly associated with heart attack/myocardial infarction in the UKBB, identified by MTWAS, UTMOST, PrediXcan, and TIGAR.** Source data are provided as a Source Data file.

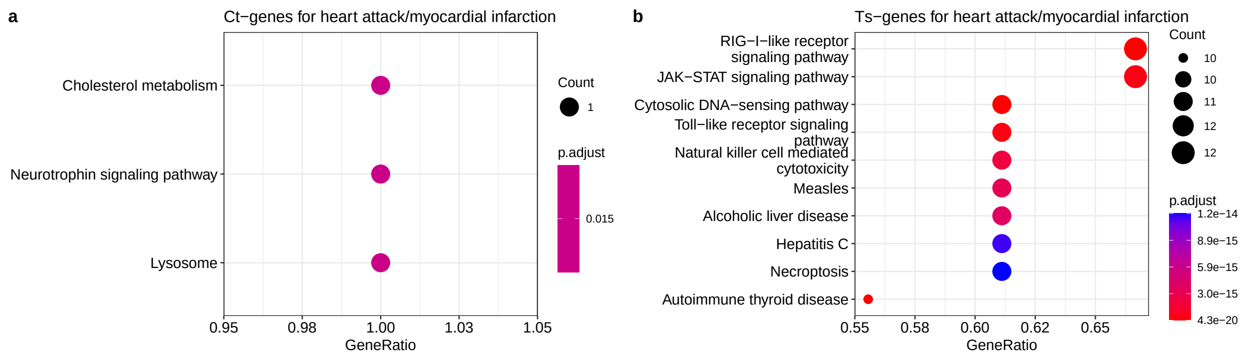

Supplementary Figure 12: **KEGG pathway enrichment analysis of the genes significantly associated with heart attack/myocardial infarction.** **a**, cross-tissue genes (genes that are regulated only by ct-eQTLs); **b**, tissue-specific genes (genes that are regulated only by ts-eQTLs) The significance of enrichment is depicted through the color of the dots, and the gene ratio is indicated by the horizontal distance between the dots and the y-axis. The sizes of the dots correspond to the gene counts. Source data are provided as a Source Data file.

2 Supplementary Tables

Supplementary Table 1: **Runtime (minutes) comparison for MTWAS and UTMOST.** Both methods were applied to 47 tissues in the GTEx dataset on chromosome 1 and chromosome 22. MTWAS first imputes the missing entries in expression matrices. Both methods include identifying eQTLs and estimating eQTL weights on gene expression. The computation was performed with an Intel Xeon processor with 2.90GHz and 128 cores.

	**MTWAS**		**UTMOST**
	Imputation	Model Training	Model Training
**chromosome 1 (1,911 genes)**	18	45	87
**chromosome 22 (401 genes)**	4	9	18

Supplementary Table 2: **The prediction accuracy of the TWAS methods MTWAS, UTMOST, PrediXcan, TIGAR, and a fine-mapping method SuSiE on GTEx whole blood tissue.** The results are based on 5-fold CV. The highest prediction R^2 and the largest number of predictable genes are highlighted in boldface.

	MTWAS	UTMOST	PrediXcan	TIGAR	SuSiE
**Mean prediction** R^2	**0.041**	0.036	0.034	0.038	0.037
**Mean signed prediction** R^2	**0.037**	0.031	0.031	0.033	0.034
# **Predictable genes** ($R^2 > 0.01$)	**10,444**	10,002	6,991	9,601	7,326
# **Predictable genes** ($FDR < 0.05$)	**6,391**	5,161	4,534	5,409	5,204

Supplementary Table 3: **The performance of MTWAS and other two settings in a replication study on the GEUVADIS cohort for lymphoblastoid cell lines.** The training weights are based on the EBV transformed lymphocytes in the GTEx datasets. The highest prediction R^2 and signed prediction R^2, and the largest number of predictable genes under the common and stringent criteria are highlighted in boldface. Setting 1: Using imputed data for identifying both ct-eQTLs and ts-eQTLs, while using only observed samples to estimate effect sizes; Setting 2: Using imputed data for identifying ct-eQTLs, while using only observed samples to identify ts-eQTL, combined with a weighting scheme for estimating effect sizes.

	**MTWAS**	**Setting 1**	**Setting 2**
**Average prediction** R^2	**0.034**	0.033	0.033
**Average signed prediction** R^2	**0.031**	0.029	0.031
# **predictable genes** ($R^2 > 0.01$)	**5,176**	5,116	5,157
# **predictable genes** ($FDR < 0.05$)	**4,339**	4,287	4,322
