# Supplementary material for: Partitioning and aggregating cross-tissue and tissue-specific genetic effects to identify gene-trait associations
Source: Nat Commun. 2024 Jul 9;15:5769. doi: 10.1038/s41467-024-49924-4 (PMC11233643; doi:10.1038/s41467-024-49924-4)
Supplement: Supplementary file 3 — Description of Additional Supplementary Files [file 41467_2024_49924_MOESM3_ESM.pdf]

Description of Additional Supplementary Files

File Name: Supplementary Data 1

Description: Summary information of the 84 UKBB self-reported cancer and non-cancer illness phenotypes with effective sample sizes larger than 5,000.

File Name: Supplementary Data 2

Description: MTWAS summary statistics of 84 UKBB phenotypes. Genes with $R^2>0.01$ in the prediction stage and adjusted $p<0.05$ (two-sided, Bonferroni correction) in the association stage were included. The eQTLs weights were trained with 47 tissues in the GTEx dataset.
