# Supplementary material for: Partitioning and aggregating cross-tissue and tissue-specific genetic effects to identify gene-trait associations
Source: Nat Commun. 2024 Jul 9;15:5769. doi: 10.1038/s41467-024-49924-4 (PMC11233643; doi:10.1038/s41467-024-49924-4)
Supplement: Supplementary file 6 — Reporting Summary [file 41467_2024_49924_MOESM6_ESM.pdf]

nature portfolio

Corresponding author(s): Lin Hou, Jun S. Liu

Last updated by author(s): May 24, 2024

Reporting Summary

Nature Portfolio wishes to improve the reproducibility of the work that we publish. This form provides structure for consistency and transparency in reporting. For further information on Nature Portfolio policies, see our Editorial Policies and the Editorial Policy Checklist.

Statistics

For all statistical analyses, confirm that the following items are present in the figure legend, table legend, main text, or Methods section.

n/a	Confirmed	
☐	☒	The exact sample size (*n*) for each experimental group/condition, given as a discrete number and unit of measurement
☐	☒	A statement on whether measurements were taken from distinct samples or whether the same sample was measured repeatedly
☐	☒	The statistical test(s) used AND whether they are one- or two-sided<br>*Only common tests should be described solely by name; describe more complex techniques in the Methods section.*
☐	☒	A description of all covariates tested
☐	☒	A description of any assumptions or corrections, such as tests of normality and adjustment for multiple comparisons
☐	☒	A full description of the statistical parameters including central tendency (e.g. means) or other basic estimates (e.g. regression coefficient) AND variation (e.g. standard deviation) or associated estimates of uncertainty (e.g. confidence intervals)
☐	☒	For null hypothesis testing, the test statistic (e.g. F, t, r) with confidence intervals, effect sizes, degrees of freedom and P value noted<br>*Give P values as exact values whenever suitable.*
☐	☒	For Bayesian analysis, information on the choice of priors and Markov chain Monte Carlo settings
☒	☐	For hierarchical and complex designs, identification of the appropriate level for tests and full reporting of outcomes
☐	☒	Estimates of effect sizes (e.g. Cohen's d, Pearson's r), indicating how they were calculated

Our web collection on statistics for biologists contains articles on many of the points above.

Software and code

Policy information about availability of computer code

Data collection	The dbGaP data were downloaded with Aspera software and the decryption was done by NCBI Decryption tool and SRA Toolkit.<br>No software was used for downloading the rest of data.
Data analysis	Data analysis was performed in R (4.3.1). All codes and the developed software are publicly available at https://github.com/szcf-weiya/MTWAS and Zenodo (https://doi.org/10.5281/zenodo.11647460).

For manuscripts utilizing custom algorithms or software that are central to the research but not yet described in published literature, software must be made available to editors and reviewers. We strongly encourage code deposition in a community repository (e.g. GitHub). See the Nature Portfolio guidelines for submitting code & software for further information.

Data

Policy information about availability of data

All manuscripts must include a data availability statement. This statement should provide the following information, where applicable:

- Accession codes, unique identifiers, or web links for publicly available datasets
- A description of any restrictions on data availability
- For clinical datasets or third party data, please ensure that the statement adheres to our policy

The genotype and gene expression data of GTEx version 8 project were downloaded from the database of Genotypes and Phenotypes (accession number phs000424.v8.p2). The genotype and gene expression data of GEUVADIS LCLs were downloaded from the EBI ArrayExpress database with accession code E-GEUV-1,

https://www.ebi.ac.uk/arrayexpress/experiments/E-GEUV-1/. The DICE project provides anonymized data for public access at https://dice-database.org. The genotype data can be accessed from the dbGaP (accession number: phs001703.v4.p1). The OneK1K single-cell gene expression and genotype data are available via Gene Expression Omnibus (GSE196830). GWAS summary statistics from the UKBB were downloaded from the repository http://www.nealelab.is/uk-biobank. The LD matrix was estimated with UKBB European ancestry samples, which can be downloaded from https://pan.ukbb.broadinstitute.org. We partitioned the genome into 1703 independent blocks using LDetect, based on 1000G reference panel with European ancestry (https://bitbucket.org/nygcresearch/ldetect-data/src/master/).

Research involving human participants, their data, or biological material

Policy information about studies with human participants or human data. See also policy information about sex, gender (identity/presentation), and sexual orientation and race, ethnicity and racism.

Reporting on sex and gender	Not relevant in this study.
Reporting on race, ethnicity, or other socially relevant groupings	Not relevant in this study.
Population characteristics	All data used in this study are genetics and gene expression data of European population.
Recruitment	N/A
Ethics oversight	The gene expression data are from GTEx Consortium, DICE, OneK1K, and GEUVADIS Project. The GWAS data are de-identified summary-statistics-level. This is a methodology-based research which does not involve data collection.

Note that full information on the approval of the study protocol must also be provided in the manuscript.

Field-specific reporting

Please select the one below that is the best fit for your research. If you are not sure, read the appropriate sections before making your selection.

☒ Life sciences ☐ Behavioural & social sciences ☐ Ecological, evolutionary & environmental sciences

For a reference copy of the document with all sections, see nature.com/documents/nr-reporting-summary-flat.pdf

Life sciences study design

All studies must disclose on these points even when the disclosure is negative.

Sample size	This is a methodology-based research. We developed a framework for gene-level test based on GWAS summary statistics. In order to discover new genes and replicate the results, we used the largest datasets available.
Data exclusions	No data were excluded.
Replication	This is a methodology-based research and has no experimental findings.
Randomization	It is not relevant to the study since it is a methodology-based research.
Blinding	It is not relevant to the study since it is a methodology-based research.

Reporting for specific materials, systems and methods

We require information from authors about some types of materials, experimental systems and methods used in many studies. Here, indicate whether each material, system or method listed is relevant to your study. If you are not sure if a list item applies to your research, read the appropriate section before selecting a response.

Materials & experimental systems

n/a	Involved in the study	
☒	☐	Antibodies
☒	☐	Eukaryotic cell lines
☒	☐	Palaeontology and archaeology
☒	☐	Animals and other organisms
☒	☐	Clinical data
☒	☐	Dual use research of concern
☒	☐	Plants

Methods

n/a	Involved in the study	
☒	☐	ChIP-seq
☒	☐	Flow cytometry
☒	☐	MRI-based neuroimaging

Plants

Seed stocks	N/A
Novel plant genotypes	N/A
Authentication	N/A
